# The effects of camelina sativa oil and high-intensity interval training on liver function and metabolic outcomes in male type 2 diabetic rats

**DOI:** 10.3389/fnut.2023.1102862

**Published:** 2023-03-01

**Authors:** Zeynab Kavyani, Parvin Dehghan, Mostafa Khani, Mousa Khalafi, Sara K. Rosenkranz

**Affiliations:** ^1^Student Research Committee, Tabriz University of Medical Sciences, Tabriz, Iran; ^2^Department of Biochemistry and Nutrition Therapy, School of Nutrition and Food Science, Tabriz University of Medical Sciences, Tabriz, Iran; ^3^Nutrition Research Center, Faculty of Nutrition and Food Science, Tabriz University of Medical Sciences, Tabriz, Iran; ^4^Faculty of Physical Education and Sport Sciences, University of Tabriz, Tabriz, Iran; ^5^Department of Physical Education and Sport Sciences, Faculty of Humanities, University of Kashan, Kashan, Iran; ^6^Department of Kinesiology and Nutrition Sciences, University of Nevada Las Vegas, Las Vegas, NV, United States

**Keywords:** camelina oil, high-intensity interval training, diabetes, inflammation and oxidative stress, hepatic steatosis

## Abstract

**Objectives:**

The purpose of this study was to evaluate the independent and combined effects of camelina sativa oil and high-intensity interval training (HIIT) on liver function, and metabolic outcomes in streptozotocin-induced diabetic rats.

**Methods:**

Forty male Wistar rats were randomly assigned to five equal groups (8 per group): Normal control (NC), diabetic control (DC), diabetic + camelina sativa oil (300 mg/kg by oral gavage per day; D + CSO), diabetic + HIIT (running on a treadmill 5 days/week for 8 weeks; D + HIIT), diabetic + camelina sativa oil + HIIT (D + CSO +  HIIT).

**Results:**

In all three intervention groups (D + CSO, D + HIIT, and D + CSO + HIIT) compared to the DC, hepatic TNF-α, MDA, and histopathology markers, decreased and hepatic PGC-1α, and PPAR-γ increased (p < 0.05). However, the effect of D + CSO was greater than D + HIIT alone. Hepatic TG decreased significantly in D + HIIT and D + CSO + HIIT compared to other groups (*p* < 0.001). Fasting plasma glucose in all three intervention groups (D + CSO, D + HIIT, and D + CSO + HIIT) and HOMA-IR in D + CSO and D + CSO + HIIT were decreased compared to DC (*p* < 0.001). Only hepatic TAC and fasting plasma insulin remained unaffected in the three diabetic groups (*p* < 0.001). Overall, D + CSO + HIIT had the largest effect on all outcomes.

**Conclusions:**

At the doses and treatment duration used in the current study, combination of CSO and HIIT was beneficial for reducing liver function and metabolic outcomes other than CSO and HIIT alone.

## Introduction

Type 2 diabetes mellitus (T2DM) is a long-term endocrine disease characterized by hyperglycemia and is associated with inflammation, oxidative stress; insulin resistance (IR), and hepatic steatosis (1, 2). There is emerging evidence that chronic hyperglycemia *via* dysregulated production of tumor necrosis factor-alpha (TNF-α), *interleukin 6* (IL-6), and C-reactive protein (CRP), along with excess free radical production and oxidative stress, plays a critical role in the development of IR and T2DM (3). Furthermore, diabetic patients have an increased risk of liver disease and liver failure, which is one of the most common causes of death in diabetic patients (4). Impaired liver function is caused by IR, oxidative stress, and inflammation in the tissue organ, and in patients with T2DM, is partly due to elevated blood glucose levels (5, 6). In addition, previous research has indicated a strong association between liver fat accumulation and T2DM, indicating an increased risk for nonalcoholic fatty liver disease (7). Therefore, the prevention of inflammation and oxidative stress leading to liver fat accumulation are therapeutic targets in patients with T2DM. Numerous studies have confirmed that consumption of omega-3 fatty acids improves both macro and micro-vascular complications of T2DM by modifying the gut microbiota (6, 8) and controlling IR (9–12), oxidative stress (13), inflammation (14, 15), lipid metabolism (16, 17), and hepatic fat deposition (18). Recently, due to current concerns about heavy metal-contaminated fish oil supplements and their adverse effects, switching omega-3 fatty acids sources from animal to plant sources has been considered (19). Camelina sativa, known as false flax, is one of the richest food sources of omega-3 fatty acids, with polyunsaturated fatty acid (PUFA) values of more than 50%, alpha-linolenic acid (ALA) 40%–45%, as well as high content of phytosterols (331–442 mg/100 g), carotenoids (103–198 mg/of carotene/kg), and tocopherols (55.8–76.1 mg/100 g) (20). According to the evidence provided by the FDA and the study conducted by Mousazadeh et al. (21), side effects have not been reported, but due to the high dose of omega-3 (more than 3 g/day), it may have gastrointestinal effects, so caution should be used in the prescription of high doses of camelina oil (22). Allong with nutrient intake (5), exercise training is an effective intervention for the treatment and prevention of metabolic disorders such as T2DM (23, 24). The effects of exercise training are associated with increased expression or activity of proteins involved in insulin signaling, subsequently modulating glycogen synthase activity, glucose transporter expression in the muscle, and improving IR, inflammation, and oxidative stress in T2DM patients (25). In a study, the benefits of strength exercise have been shown in reducing hepatic triglyceride content among T2DM rats (26). Traditionally, moderate-intensity continuous training has been considered an effective method of training for improving health outcomes in T2DM patients; however, high-intensity interval training (HIIT) is a well-accepted alternative strategy that may serve as a way for some individuals to save time (27).

Peroxisome proliferator-activated receptor gamma (PPAR-γ) controls fatty acid, glucose, and inflammatory processes (28, 29). PPAR-γ agonists directly activate liver glucose-sensing genes, improving glucose homeostasis and insulin sensitivity in T2DM patients (30). Omega-3 fatty acids and exercise upregulate PPAR-γ coactivator 1α (PGC-1α), which regulates mitochondrial biogenesis and activates PPAR-γ (31, 32). HIIT is an effective approach for reducing lipogenesis (33) and improving inflammation (34), IR, postprandial glycemia (35) fat loss (36), visceral fat and liver fat (37) which are all important treatment targets for patients with T2DM. To the best of our knowledge, there is no study investigating the simultaneous effects of CSO intake and HIIT on liver function, the status of inflammation, oxidative stress, and lipogenesis. The current intervention study aimed to examine to determine the independent and combined effects of camelina sativa oil and HIIT on liver function, and metabolic outcomes in male T2DM rats. We hypothesized that the combination of both would provide superior benefits for reducing inflammation, oxidative stress and lipogenesis, as well as liver triglycerides.

## Materials and methods

### Ethics statement

All animal experiments were carried out in accordance with the National Institutes of Health’s ethical standards for the care and use of laboratory animals (NIH; Publication No. 85-23, revised 1985), which were examined and confirmed by the Veterinary Ethics Committee of Tabriz University of Medical Sciences (Approval No.: IR.TBZMED.AEC.1401.040).

### Experimental design

**Figure 1:**
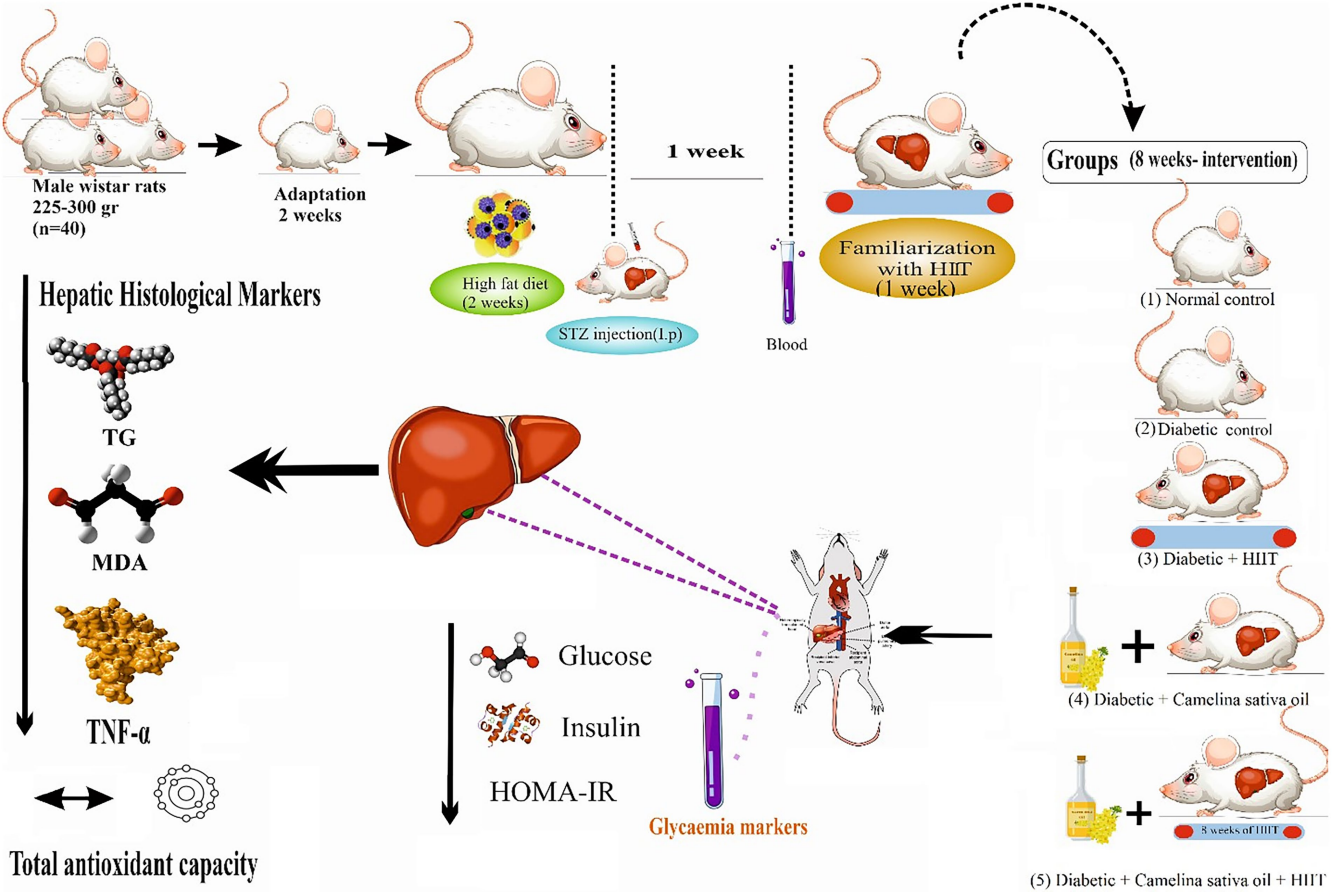
Experimental design. HIIT, High-intensity interval training.

Forty (3-month-old) adult male Wistar rats (225–300 g), were obtained from the Central Animal House, Tehran University of Medical Sciences, and adapted to the experimental conditions in standard polypropylene cages (4 rats/cage) under controlled humidity (50 ± 5%) and temperature (20 ± 2°C) with a 12 h light/dark cycle for 2 weeks. T2DM was induced through a combination of a high-fat diet (HFD) and a single dose of Streptozotocin (STZ; 35 mg/kg, intraperitoneal (ip) 0.1 M citrate buffer, pH 4.5) after rats have fasted for 5 h. Rats were fed with a high-fat diet (45% fat, 34% carbohydrate, and 21% protein) prepared from animal tail oil (450 g per 100 g standard pellet) and cholesterol gel for an initial period of 2 weeks and then injected intraperitoneally with a single dose of streptozotocin (STZ, 35 mg/kg of body weight), which was freshly prepared by dissolving in 0.1 M citrate buffer (pH 4.5). A week after induction of T2DM, the rats with blood glucose levels of 250 mg/dL or greater were considered diabetic (38). Blood glucose levels were measured by a glucometer from the tail vein of animals after a 12 h fast following the 2-week high-fat diet. Animals had access to *ad libitum* water and standard chow (54% carbohydrate, 26% protein, 13% fat, 5% fiber, and 3% vitamins, and minerals). Rats were randomly allocated into five groups (8 per group, calculated using G*Power) including 1-Normal control (NC) was given normal saline by oral gavage; 2-Diabetic control (DC) was given normal saline by oral gavage; 3-Diabetic + camelina sativa oil (300 mg/kg) by oral gavage (D + CSO), 4-Diabetic + HIIT (D + HIIT) were given normal saline by oral gavage, and 5-Diabetic + camelina sativa oil (300 mg/kg) by oral gavage + HIIT (D + CSO + HIIT) (Figure 1).

### Camelina oil supplementation

We used a gas chromatograph to analyze the fatty acid composition of CSO (Bistun Shafa Co, Kermanshah, Iran). The study’s CSO analysis showed that the highest fatty acids were linolenic acid (29.70%), linoleic acid (21.03%), and oleic acid (16.41%; Table 1; 39). According to the evidence provided by the FDA, CSO has been deemed to be generally safe, and is registered as food oil in many European nations. Rats in the CSO conditions were fed by oral gavage based on weight at a dose of 300 mg/kg per day for 8 weeks. Rats in the non-CSO groups were given saline, % 0.9 NaCl, *via* oral gavage (1 mL per day). Oral gavages were performed before exercise in the HIIT conditions (40).

**Table 1:** Composition of fatty acids present in camelina sativa oil.

Fat	%	Fatty acid	Name	Camelina oil (%)
SFA	13.82	C12:0	Lauric acid	0.00
		C14:0	Myristic acid	0.09
		C16:0	Palmitic acid	6.45
		C18:0	Stearic acid	2.56
		C20:0	Arashidic acid	1.89
		C21:0	Heneicosanoic acid	1.66
		C22:0	Behenic acid	1.00
		C24:0	Lignoseric acid	0.17
MUFA	34.36	C16:0	Palmitoleic acid	0.17
		C18:0	Oleic acid	16.41
		C20:0	Elcosenoic acid	14.09
		C22:0	Erucic acid	3.21
		C24:0	Nervonic acid	0.47
PUFA	51.83	C18:0	Linoleic acid	21.03
		C18:0	Linolenic acid	29.70
		C20:0	Elcosadienoic acid	0.68
		C20:0	Elcosatrienoic acid	0.41

SFA, Saturated fatty acids; MUFA, Monounsaturated fatty; PUFA, Polyunsaturated fatty acids.

### Exercise training protocol

Before the interventions, all rats were familiarized with treadmill running for 1 week (10 min per day) at a speed of 8–10 m per min with a 0% incline. Afterward, HIIT was performed 5 days per week for 8 weeks on a treadmill at 6 p.m. (lights off). The HIIT program involved 8 sets of 3 min of high-intensity running at 85%–90% of one’s maximum speed, followed by 2 min of active rest at 30%–40% of one’s maximum speed. The HIIT protocol comprised 5-min warm-up and cool-down intervals that were low-intensity (30%–40% of maximum speed) before and after each session. To determine the maximal speed at the time of maximum oxygen consumption (VO2 max), rats ran 5 m on a treadmill at a speed of 6 m/min with a zero-degree gradient for 5 min (warm-up), and then the treadmill speed increased to 3 m/min every 3 min until the animals reached the point of extinction and could no longer continue. The incapacity of the rats to continue the workout program with increasing speed and collision three times in 1 min to the end of the treadmill was the criterion for reaching VO2 max, hence VO2 max was assessed using speed. Every 2 weeks, the animals were assessed, and the training intensity was determined based on the new test values.

### Experimental procedures

After fasting for 12–14 h, and 48 h after the previous training session, all rats were sedated with a painless intraperitoneal injection of ketamine (90 mg/kg) and zailazin (10 mg/kg). Blood was collected from the tail vein; plasma was separated *via* centrifuging at 3,500 rpm for 5 min. After blood sampling, the animals were sacrificed and the livers were removed. The liver tissue samples and serum samples were flash-frozen and stored at −70°C, and the remaining livers were used for histopathological study and were homogenized in appropriate buffers for analysis of biochemical parameters like inflammatory and oxidative stress indices of the liver. Based on the aim of present study, markers of liver function, inflammation and oxidative stress were the main outcomes and glycemia markers and hepatic histopathology were secondary outcomes.

### Measurement of hepatic TNF-α

TNF-α levels were measured using an ELISA kit (catalog no. DY510-05, R&D System) after liver tissue aliquots were homogenized in accordance with the manufacturer’s instructions. All TNF-α analysis were carried out in duplicate serial dilutions.

### Measurement of hepatic MDA and TAC

The presence of malondialdehyde (MDA), a sign of lipid peroxidation, was measured. In a nutshell, livers were treated as previously reported after being homogenized in a solution of 1.15% KCl (26). By comparing the OD550 of the reference solutions of 1,1,3,3-tetramethoxypropan 99% malondialdehyde bis (dymethyl acetal) 99% (Sigma), the sample absorbance was determined by spectrophotometry, and MDA values were derived (41). A decrease in the production of thiobarbituric acid reactive compounds served as the basis for measuring the hepatic total antioxidant capacity (TAC) (42). A commercially available colorimetric kit (Bioquochem FRAP Assay Kit, KF-01-003, R&D System) was used to measure the hepatic TAC levels in accordance with the manufacturer’s recommendations. Results were adjusted for protein levels (43).

### Measurement of hepatic TG

Hepatic triglyceride (TG) concentrations were measured using commercially available colorimetric kits (Triglyceride G-Test kit, Wako Pure Chemical Industries) according to manufacturer instructions.

### Hepatic PGC-1α and PPAR-γ

Western Blotting methods were used to messure protein levels of hepatic PGC-1α and PPAR-γ. Protein lysates were isolated from 500 mg of liver tissue in lysis buffer (500 μL Tris, PH = 8, 150 mM sodium chloride, 1% NP-40, 0.5% sodium deoxycholate, 0.1% SDS, and 0.1 mM EDTA) supplemented with a complete protease inhibitor cocktail and centrifuged at 12,000*g* for 10 min at 4°C. The Bradford method was used to determine the protein concentration in the supernatant (44). Proteins were separated using SDS-polyacrylamide gel electrophoresis with 8%–12% denatured ready gel (Bio-Rad, Hercules, CA, United States) and transferred to a PVDF membrane (Roche, West Sussex, United Kingdom). To prevent nonspecific bindings, the membrane was blocked for 1 h in 5% BSA in tris-buffered saline and 0.1% Tween 20 (TBST). Blots were then incubated overnight at 4^°^ C with the following primary antibodies: β-actin (sc-47,778, 1: 300), PPAR-γ (ab20935), and PGC-1α (ab54481), all purchased from Cell Signaling Technology. The membrane was then washed three times and incubated for 1 h at room temperature in 5% milk in TBST with the appropriate secondary antibody (m-IgG BP-HRP: sc-516,102, and mouse anti-rabbit IgG-HRP: sc-2,357) (44). Protein bands were visualized using an enhanced chemiluminescence (ECL) reagent and quantified using densitometry analysis with Image J software.

### Liver histopathological study

After being cleaned with normal saline, liver tissues were fixed in 10% buffered formalin for 48 h. For the purpose of the histological evaluation, samples were embedded in paraffin, divided into 5-lm pieces, stained with hematoxylin and eosin (H and E), and examined under a light microscope. Scores were made in 10 fields of each H and E-stained slide, which were then examined under a light microscope to determine the liver’s histological, hydropic degeneration, microvesicular and macrovesicular vacuoles, sinusoidal congestion, and cell necrosis findings (40). Scores for the histopathological results were none (−), mild (+), moderate (++), and severe damage (+++) (45, 46).

### Measurement of fasting blood glucose, insulin, HOMA-IR

Fasting blood glucose was measured by using commercially available colorimetric diagnostic kits (Pars Azmoon kit, Iran) according to the instructions. The level of insulin was determined using the rat Insulin ELISA Kit (ALPCO, Catalog no: 80-INSRTH-E01). HOMA-IR was employed to assess the IR *via* the following formula (47):


$$\mathrm{HOMA}-IR=\left[\begin{array}{l} \mathrm{fasting insulin}\;\left(\mathrm{mU}/L\right)\\ \times \mathrm{fasting blood glucose}\;\left(\mathrm{mg}/\mathrm{dL}\right) \end{array}\right]/405$$

### Statistical analysis

The Shapiro–Wilk test was used to assess the distribution’s normality. The variances were then shown to be homogenous by a Leven test. The mean differences between the groups were examined using a one-way analysis of variance (ANOVA). Using Tukey’s Test, differences between two groups were measured. Means and SEM were used to express the data. Statistical significance was defined as a value of *p* < 0.05. Pearson correlation coefficients were used to ascertain the relationship between the variables. The statistical software SPSS was used for all calculations (Version 20.00).

## Results

### Hepatic TNF-α

**Figure 2:**
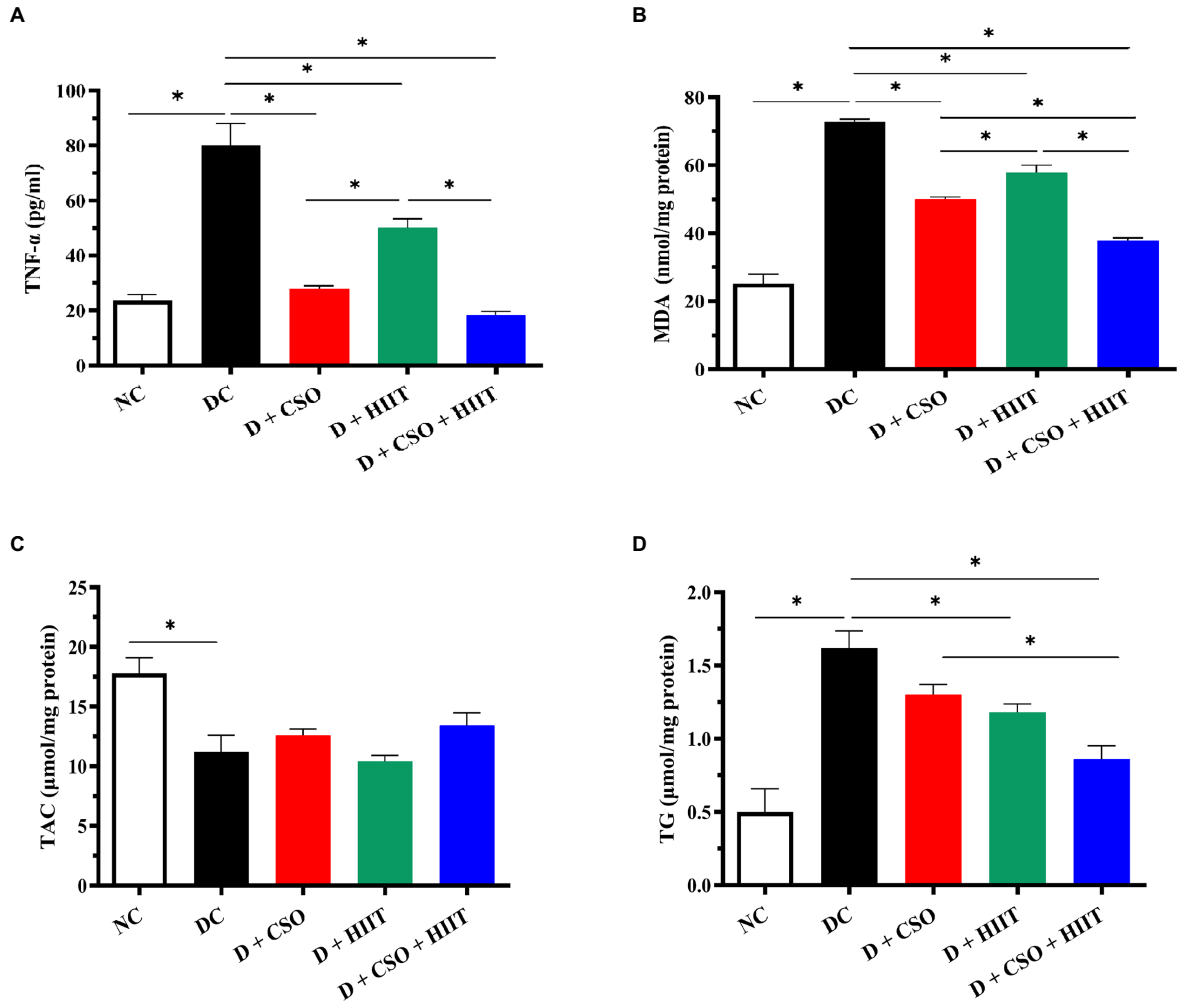
The effect of D, D + CSO, D + HIIT, and D + CSO + HIIT on hepatic **(A)** TNF-α, **(B)** MDA, **(C)** TAC, and **(D)** TG. One-way ANOVA followed by Tukey post-test was used. Data are represented as means ± SEM and significant differences between groups are indicated by *p < 0.05. NC, Normal control; DC, Diabetic control; D + CSO, Diabetic + camelina sativa oil; D + HIIT, Diabetic + HIIT; D + CSO + HIIT, Diabetic + camelina sativa oil + HIIT.

Hepatic TNF-α was significantly (*p* < 0.001, Figure 2A) increased in the DC group compared to NC. In contrast, TNF-α decreased in all three intervention groups as compared to DC, an effect that was greater in D + CSO and D + CSO + HIIT as compared to D + HIIT alone.

### Hepatic MDA and TAC

Hepatic MDA increased in the DC group compared to NC (*p* < 0.001, Figure 2B). Hepatic MDA decreased in all three intervention groups compared to DC (*p* < 0.001). These decreases were greater with D + CSO and D + CSO + HIIT compared to D + HIIT. In addition, D + CSO + HIIT significantly decreased hepatic MDA compared to D + CSO alone. Hepatic TAC decreased in the DC group compared to NC; however, it did not change significantly (*p* > 0.05, Figure 2C) with the three interventions.

### Hepatic TG

Hepatic TG increased in the DC group compared to NC (*p* < 0.001, Figure 2D), and decreased significantly in D + HIIT and D + CSO + HIIT compared to DC. In addition, Hepatic TG decreased in D + CSO + HIIT compared to D + CSO alone. Changes in D + CSO alone were not statistically significant as compared with DC (*p* > 0.05).

### Hepatic PGC-1α and PPAR-γ

**Figure 3:**
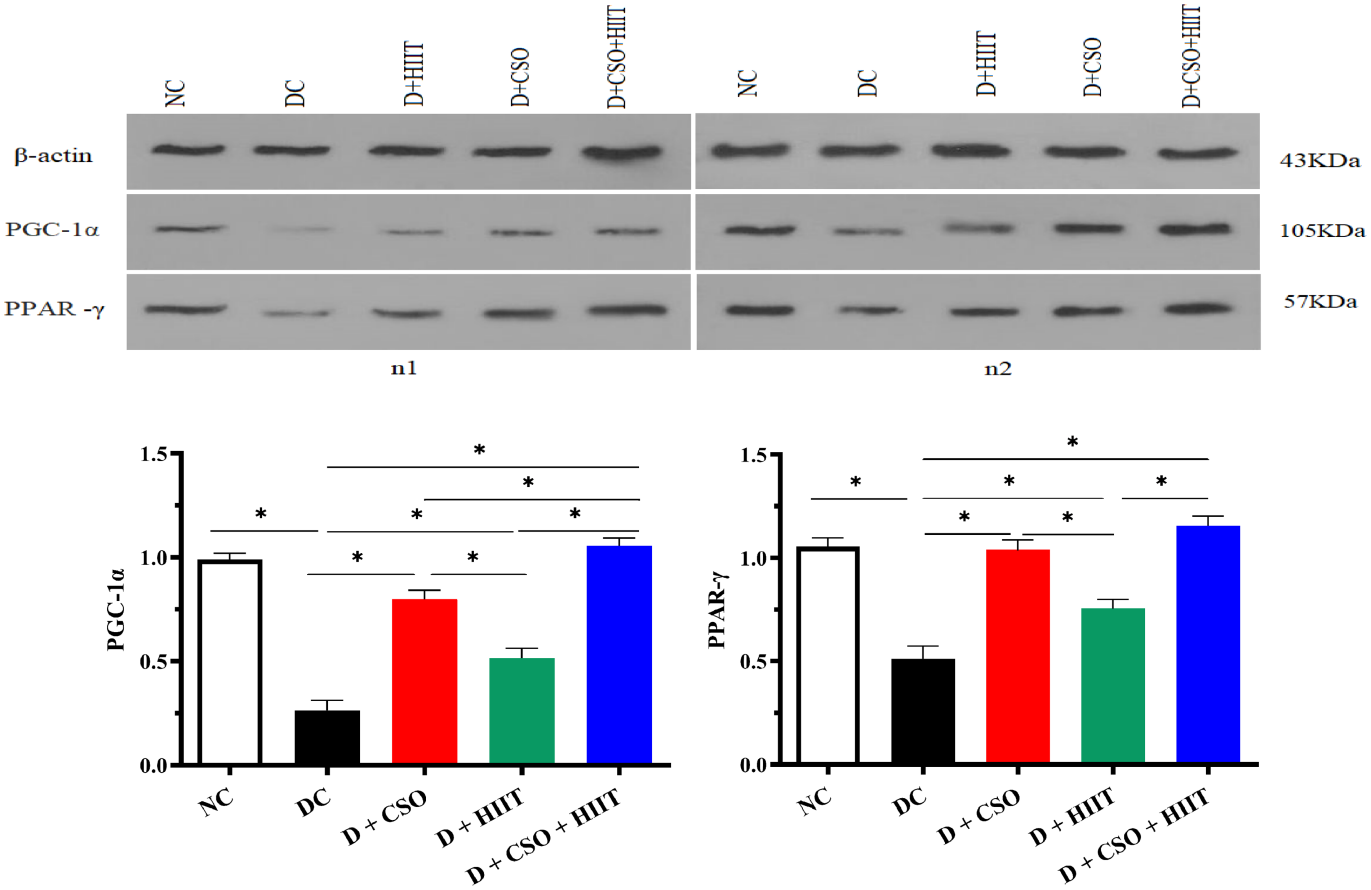
Western bloting analysis of protein expression of β-actin, PGC-1α, and PPARy. The effect of D, D + CSO, D + HIIT, and D + CSO + HIIT on PGC-1α, and PPARy. NC, Normal control; DC, Diabetic control; D + CSO, Diabetic + camelina sativa oil; D + HIIT, Diabetic + HIIT; D + CSO + HIIT, Diabetic + camelina sativa oil + HIIT.

Hepatic PGC-1α and PPAR-γ decreased in the DC group compared to NC. In contrast, both PGC-1α and PPAR-γ increased in all three intervention groups as compared to DC. For PGC-1α, D + CSO + HIIT significantly increased hepatic PGC-1α as compared to D + HIIT alone (*p* < 0.001) and D + CSO alone (*p* < 0.001). In addition, D + CSO significantly increased hepatic PGC-1α as compared to D + HIIT alone (*p* < 0.001). For PPAR-γ, D + CSO + HIIT and D + CSO alone significantly increased hepatic PPAR-γ as compared to D + HIIT alone (*p* < 0.001 and *p* < 0.05, respectively). But there was not a significant difference between D + CSO + HIIT and D + CSO alone (*p* > 0.05; Figure 3).

### Hepatic histopathology

**Figure 4:**
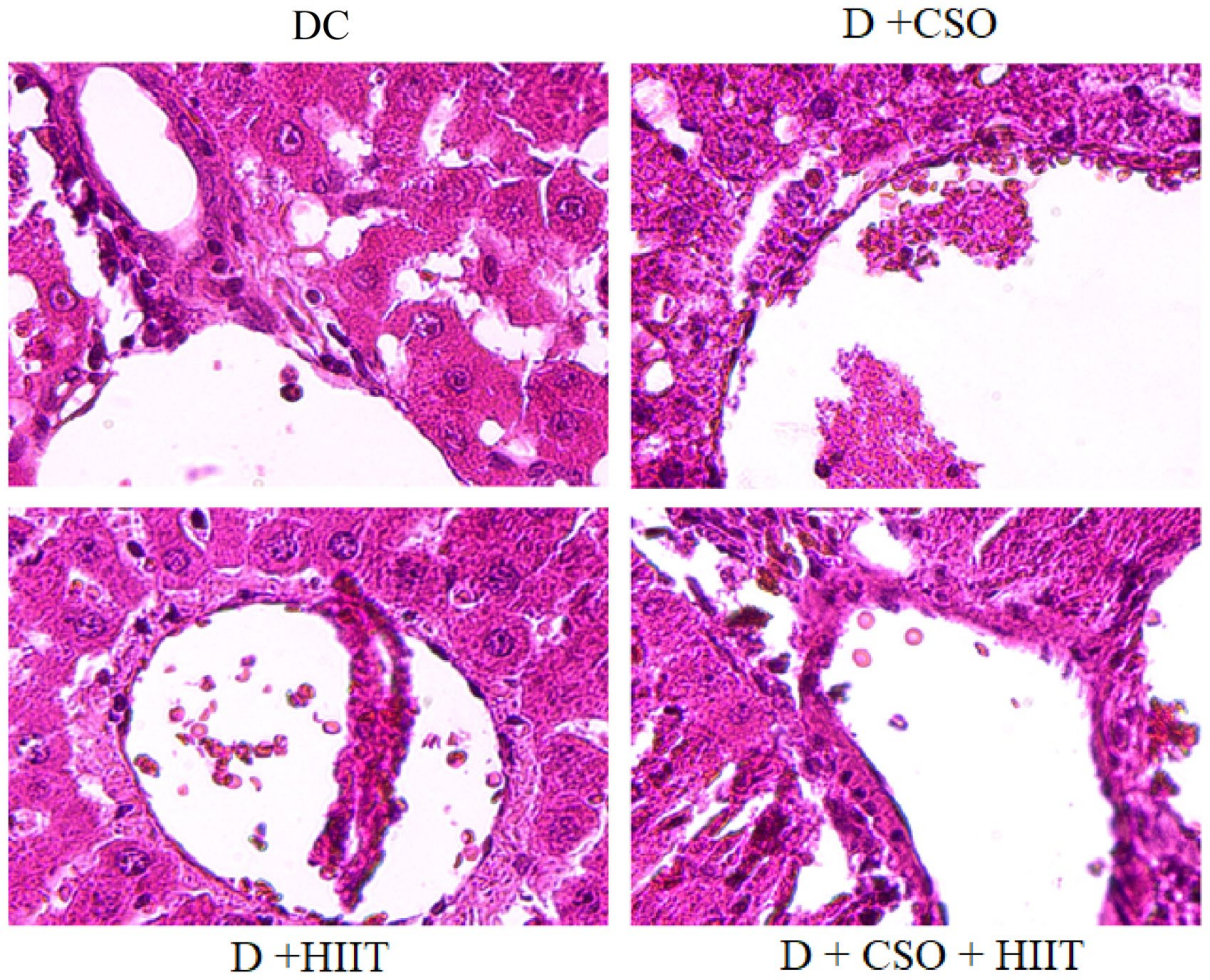
The effect of DC, D + CSO, D + HIIT, and D + CSO + HIIT on hepatic histopathology, 400 × magnification, DC, Diabetic control; D + CSO, Diabetic + camelina sativa oil; D + HIIT, Diabetic + HIIT; D + CSO + HIIT, Diabetic + camelina sativa oil + HIIT.

Hepatic histopathology markers including hydropic degeneration, micro-vesicular vacuoles, macro-vesicular vacuoles, and sinusoidal congestion were increased in the DC group compared to NC, whereas these markers were decreased in all three intervention groups compared to DC (Table 2). These decreases were more significant in D + CSO + HIIT as compared with D + CSO and D+ HIIT alone (Figure 4).

**Table 2:** The effect of camelina sativa oil and HIIT on changes in liver histopathology of male type 2 diabetic rats.

Groups	Hydropic degeneration	Microvesicular vacuoles	Macrovesicular vacuoles	Sinusoidal congestion	Cell necrosis
NC	0	0	0	0	0
DC	3+	3+	3+	2+	3+
D + HIIT	2+	2+	3+	3+	2+
D + CSO	2+	2+	2+	2+	1+
D + CSO + HIIT	0	1+	1+	1+	0

NC, normal control; DC, diabetic control; D + CSO, diabetic + camelina sativa oil; D + HIIT, Diabetic + HIIT; D + CSO + HIIT, Diabetic + camelina sativa oil + HIIT.

### Glycaemia markers

**Figure 5:**
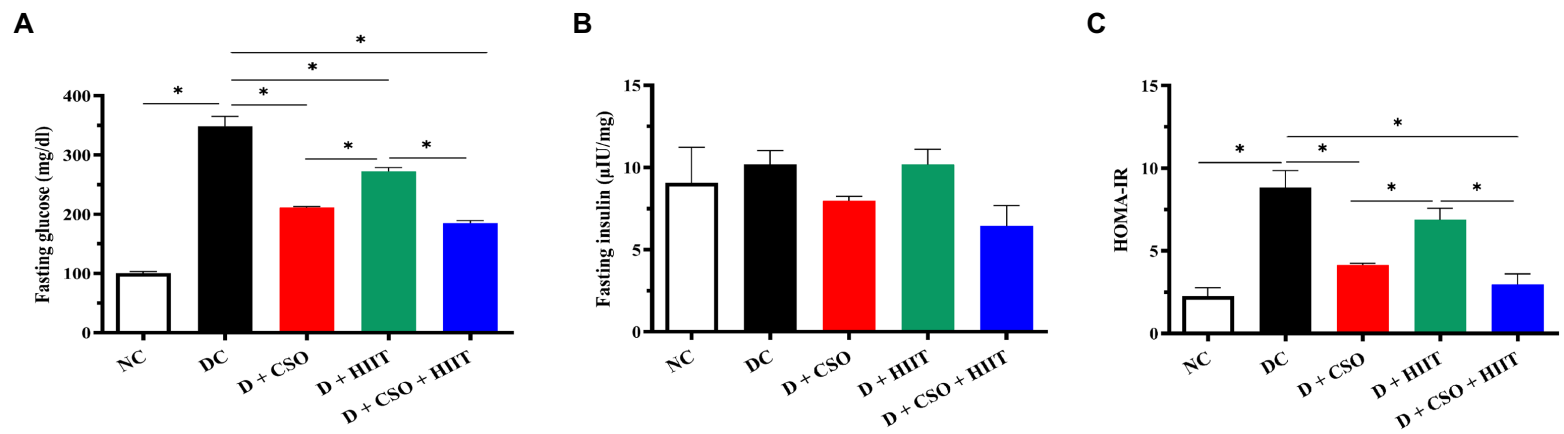
The effect of D, D + CSO, D + HIIT, and D + CSO + HIIT on glycemia markers including **(A)** glucose, **(B)** insulin, and **(C)** HOMA-IR. One-way ANOVA followed by Tukey post-test was used. Data are represented as means ± SEM and significant differences between groups are indicated by *p < 0.05. NC, Normal control; DC, Diabetic control; D + CSO, Diabetic + camelina sativa oil; D + HIIT, Diabetic + HIIT; D + c + HIIT, Diabetic + camelina sativa oil + HIIT.

Fasting plasma glucose and HOMA-IR were significantly (*p* < 0.001, Figure 5A; *p* < 0.001, Figure 5C) increased in diabetic rats compared to NC. In contrast, fasting plasma glucose in all three interventions (D + CSO, D + HIIT, and D + CSO + HIIT) and HOMA-IR in D + CSO and D + CSO + HIIT were decreased compared to DC. In addition, D + CSO+ HIIT significantly (*p* < 0.001) decreased fasting glucose and HOMA-IR compared to D + HIIT alone (*p* < 0.001). Also, D+CSO significantly decreased fasting plasma glucose and HOMA-IR compared to D+HIIT (*p* < 0.001). However, fasting plasma insulin changes were not significantly (*p* > 0.05, Figure 5B) different between groups.

## Discussion

There has been some previous research examining the independent effects of CSO and HIIT on glycemic control (5, 48, 49); however, there is no previously published study on the combined effects of CSO and HIIT, in particular in a T2DM model or in patients with T2DM. Therefore, in the present study, the combined effects of CSO and HIIT on glycemic indices, inflammatory and oxidative stress markers in hepatic cells, hepatic triglyceride content, and liver histopathological findings were investigated in male T2DM rats. According to our findings, there were improving synergistic effects of CSO and HIIT for 8 weeks on glucose, HOMA-IR, hepatic MDA, TNF-α, TG, PPAR-γ, PGC-1α and histopathology markers; however, insulin and TAC did not change significantly in three intervention groups.

Our results suggest that CSO, as a rich source of omega-3 fatty acids exerted positive effects on glycemic and insulin resistance markers, in agreement with previous research in patients with NAFLD (5, 6) and impaired glucose metabolism (48). The proposed anti-hyperglycemic mechanisms of action by which CSO may influence insulin resistance are mostly related to its omega-3 fatty acids contents. Omega-3 fatty acids are thought to improve insulin resistance by modulating mitochondrial bioenergetics and endoplasmic reticulum stress, and through upregulation of PPAR-γ, one of the main regulators of glucose homeostasis (50, 51). Moreover, HIIT improves insulin resistance by increasing mitochondrial biogenesis, GLUT-4 translocation, and PGC-1α (52). Therefore, the synergistic effects of CSO and HIIT on glycemic parameters might be related to their shared effects on mitochondrial bioenergetics, PPAR-γ activity, and GLUT-4 translocation. Our findings showed that CSO increased PPAR-γ protein expression. In line with our findings, Taranu et al. and Tejera et al., using *in-vivo* models, reported that ω-3 PUFA rich CSO increased PPAR-γ expression (53, 54). Moreover, ω-3 PUFAs have been recognized as the natural agonists of PPAR-γ (55). The current study, for the first time, evaluated the effects of CSO plus HIIT on PPAR-γ protein expression in an animal model. However, our study did not show a beneficial effect for HIIT on PPAR-γ. Similar result were obtained from another *in-vivo* investigation (56). However, another long-term study (12 weeks) in rats reported that HIIT led to a significant increase in PPAR-γ expression following a high-fat diet (57). Therefore, additional studies with longer durations may show a synergistic effect for CSO and HIIT on PPAR-γ expression. This synergistic effect for CSO and HIIT on PGC-1α protein expression was shown for the first time in our study. Additional studies are needed to elucidate other anti-hyperglycemic machanisms of action of HIIT plus CSO, including their possible synergistic effects on GLUT4 translocation and mitochondrial bioenergetics.

Only a few previous studies have investigated the anti-oxidant and anti-inflammatory properties of CSO. In agreement with the current study results, Kavyani et al. showed that co-supplementation of CSO and prebiotics for 12 weeks led to significant decreases in MDA and hs-CRP, and increases in TAC among patients with NAFLD (6). Musazadeh et al. reported similar results with CSO plus a calorie-restricted diet for 12 weeks in patients with NAFLD (5). An *in-vivo* study showed that CSO supplementation led to significant increases in the activity of anti-oxidant enzymes along with significant decreases in MDA levels (58). Regarding TAC, our study results conflict with this previous evidence, and the differences between the current TAC results and previous results may be related to the duration of supplementation (12 weeks vs. 8 weeks). Kavyani et al. demonstrated anti-inflammatory properties of omega-3 fatty acids (15). It is well established that oxidative stress plays a key role in the pathophysiology of insulin resistance and T2DM (59, 60), and there is an increasing body of evidence from animal studies confirming oxidative stress-induced insulin resistance and the improvement in insulin signal transduction and glucose homeostasis through use of antioxidants (61–63).

Omega-3 fatty acids can modulate immune system function (64) and the production of pro-inflammatory cytokines (65). Moreover, omega-3 fatty acids are natural PPAR-γ agonists and can inhibit Nuclear Factor-Kappa-B (NF-ĸB) activity, the main modulator of inflammatory cascades (66). The anti-oxidant effects of omega-3 fatty acids are mainly related to changes in cellular membrane structures leading to decreases in lipid peroxidation (13). Moreover, other compounds in CSO such as phytosterols, carotenoids, and tocopherols contribute to its anti-oxidant effects (5, 20).

It’s been hypothesized that activities that increase oxygen consumption can increase free radicals and oxidative stress (67). Acutely, HIIT induces oxidative stress and lipid peroxidation by increasing NADPH oxidase, xanthine oxidase, phospholipase A2 activity, mitochondrial cytochrome c, and catecholamine oxidation (68, 69). However, with chronic exercise training, there are adaptive mechanisms that contribute to the reduction of oxidative stress, including the upregulation of redox signaling cascades and endogenous antioxidant enzymes, muscle hypertrophy, glucose uptake by skeletal muscle, and mitochondrial biogenesis (70). However, co-supplementation with an antioxidant-rich source such as CSO is necessary to accelerate the balance of oxidative stress induced by HIIT.

Beneficial synergistic effects for D + CSO + HIIT on hepatic TG, hepatic histopathology, and expression of PGC-1α were demonstrated in the current study. Previous studies have suggested that HIIT performed for 12 weeks significantly reduces intrahepatic lipid levels (71, 72). However, Winn et al. showed that the reduction of intrahepatic lipid levels did not significantly differ between different exercise intensities after 4 weeks (73). Similarly to our study, Kamal et al. investigated the effects of an 8-week HIIT program and found that HIIT was effective in decreasing intrahepatic lipid levels. However, most study participants received metformin, which can also have beneficial effects on hepatic fat levels (74). Also in agreement with the current results, hepatic histopathology examination in an *in-vivo* study revealed that 8 weeks of HIIT improved liver function (75). The current results showed that HIIT for 8 weeks can be beneficial in improving hepatic triglyceride levels.

In terms of hepatoprotective effects of CSO, Musazadeh et al. in a clinical trial study on NAFLD patients showed that CSO supplementation led to a significant decrease in alanine aminotransferase, an enzyme indicating a poor liver function in high levels. However, other liver enzymes did not significantly differ between CSO and placebo groups (21). A previous narrative review reported that improvement in hepatic steatosis and liver function following HIIT was associated with improved liver mitochondrial function, increased hepatic PPAR-α, and PPAR-γ content, improved insulin sensitivity, and suppression of hepatic *de novo* lipogenesis (76). The cellular mechanisms responsible for the positive effects of CSO on liver function have not been fully elucidated. However, anti-inflammatory, antioxidant, and anti-hyperlipidemia effects, and regulation of glucose homeostasis have been suggested. Also, the effects of omega-3 fatty acids on liver function have been investigated in previous studies (77, 78). Other plant-based omega-3 fatty acid sources such as flax seed (79), walnut (80), or chia (81) exerted hepatoprotective effects.

The current study is the first to investigate the synergistic effects of CSO and HIIT on glycemic, inflammatory, oxidative stress, and total antioxidant capacity biomarkers and liver function in an animal model of T2DM.

Some limitations should be considered when interpreting results. First, the overall treatment duration was short as compared to some similar studies. However, our results were in agreement with most studies of longer durations. Second, other biomarkers of inflammation, oxidative stress, and liver function were not included. For example, various interleukins, hs-CRP, antioxidant enzymes, aspartate aminotransferase (AST), and alanine transaminase (ALT) were not studied. Results may have differed if we had used other or additional biomarkers, in particular, antioxidant capacity should be further elucidated. Of course, a rat model of T2DM does not necessarily generalize to human participants with T2DM. Therefore, further studies in both animal and human models should be conducted to clarify all aspects of the effects of CSO and HIIT in type 2 diabetes. However, the strengths of our study should also be mentioned. Our study was the first to evaluate the synergistic effects of CSO plus HIIT on liver function, and metabolic outcomes, as well as glycemic markers in an animal model of T2DM. In addition, Western-blotting as an accurate method was performed to reach a more accurate conclusion of the antihyperglycemic mechanisms of CSO plus HIIT. Whereas, most similar studies assessed gene expression with real-time PCR methods. The current study also investigated various biomarkers to obtain a more comprehensive picture of the beneficial effects of CSO plus HIIT on T2DM to pave the way for future clinical trials.

## Conclusion

The current study indicated that CSO and HIIT, independently and combined, exerted beneficial effects on fasting blood glucose, HOMA-IR, hepatic TNF-α, MDA, TG, PPAR-γ, PGC-1α, and histopathology markers. Only hepatic TAC and fasting plasma insulin remained unaffected in all the three interventions groups. However, combination of CSO and HIIT had the largest effect on liver function and metabolic outcomes other than CSO and HIIT alone.

## Data availability statement

The original contributions presented in the study are included in the article/supplementary material, further inquiries can be directed to the corresponding author.

## Ethics statement

The animal study was reviewed and approved by the Veterinary Ethics Committee of Tabriz University of Medical Sciences (approval no.: IR.TBZMED.AEC.1401.040). Written informed consent was obtained from the owners for the participation of their animals in this study.

## Author contributions

ZK: drafting of the manuscript, acquisition of data, and approval of the article. PD: contributions to concept/design, data analysis/interpretation, critical revision of the manuscript, and approval of the article. MosK: contributions to implementation of the study, critical revision of the manuscript, and approval of the article. MouK: contributions to data analysis/interpretation, critical revision of the manuscript, and approval of the article. SR: contributions to design of the study, critical revision of the manuscript, and approval of the article. All authors contributed to the article and approved the submitted version.

## Funding

This study was funded by the Vice-chancellor for Research and Student Research Committee of Tabriz University of Medical Sciences, Tabriz, Iran (grant number: 69899).

## Conflict of interest

The authors declare that the research was conducted in the absence of any commercial or financial relationships that could be construed as a potential conflict of interest.

## Publisher’s note

All claims expressed in this article are solely those of the authors and do not necessarily represent those of their affiliated organizations, or those of the publisher, the editors and the reviewers. Any product that may be evaluated in this article, or claim that may be made by its manufacturer, is not guaranteed or endorsed by the publisher.
